# Supplementary material for: Engineering protein-protein devices for multilayered regulation of mRNA translation using orthogonal proteases in mammalian cells
Source: Nat Commun. 2018 Oct 22;9:4392. doi: 10.1038/s41467-018-06825-7 (PMC6197189; doi:10.1038/s41467-018-06825-7)
Supplement: Supplementary file 1 — Supplementary Information [file 41467_2018_6825_MOESM1_ESM.pdf]

Supplementary Information

Engineering protein-protein devices for multilayered regulation of mRNA translation using orthogonal proteases in mammalian cells

Cella F. et al.

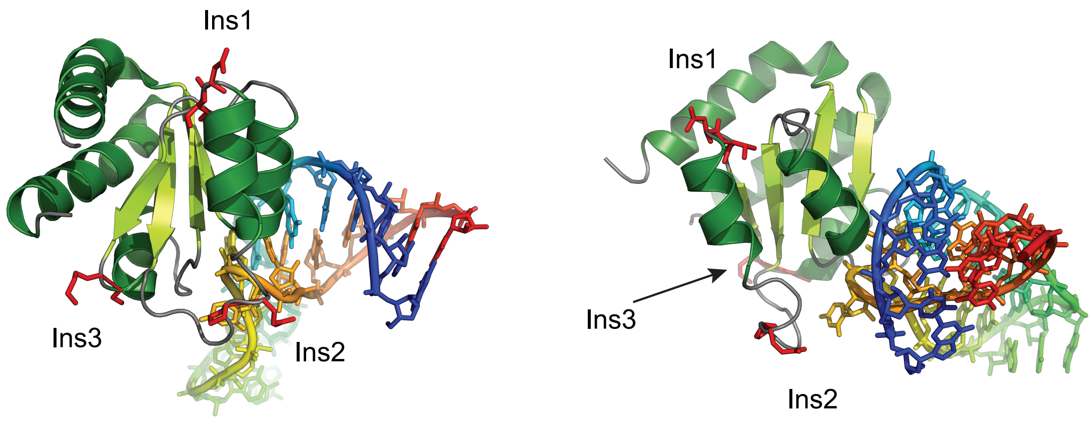

Supplementary Figure 1. Alternative views of the L7Ae/K-turn structure showing TCS insertion sites. Protein structure was determined in[1] (PDB id: 1RLG). L7Ae-TCS structure visualization was performed with pymol[2]. TCS insertion sites in L7Ae were placed after amino acid residue N70 (L7AeCS1), P56 (L7AeCS2), or K77 (L7AeCS3), shown in red for each side.

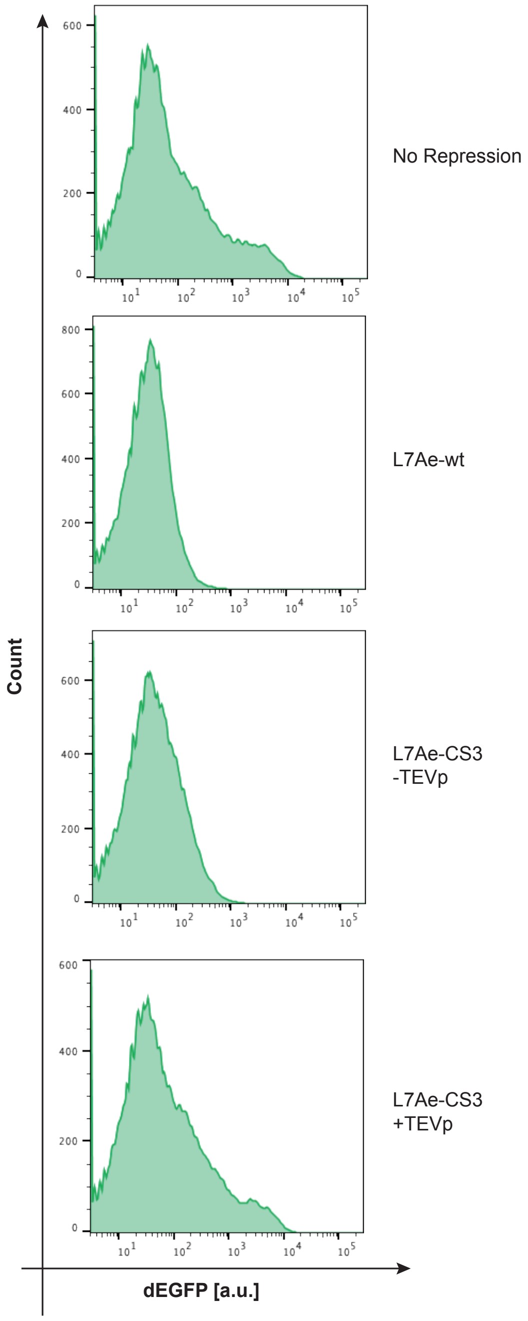

Supplementary Figure 2. Representative flow cytometry histograms of HEK293 cells tested with L7Ae-CS3 with TEVp cleavage site inserted after amino acid residue K77. A destabilized EGFP (dEGFP) with 2Kt motifs in the 5'UTR is translated in the absence of the RBP (No Repression), whereas is repressed in the presence of wild type L7Ae (L7Ae-wt) or L7Ae-CS3 (L7Ae-CS3 -TEVp). TEVp expression down-regulates L7AeCS3 activity, thus rescuing EGFP translation (L7Ae-CS3 +TEVp).

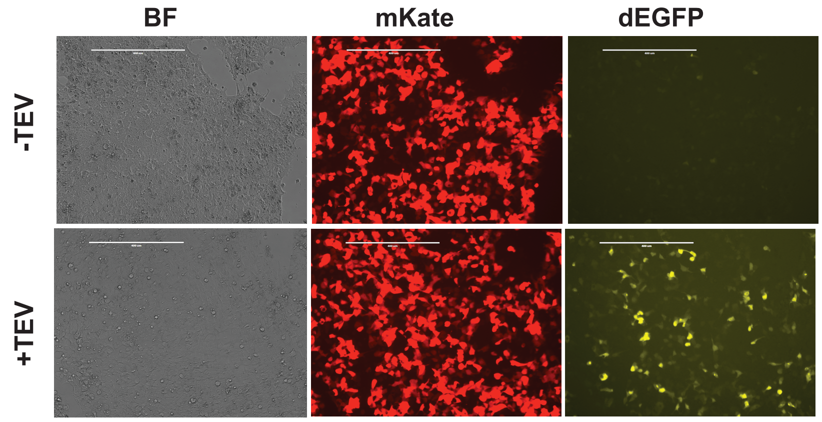

Supplementary Figure 3. Microscopy images for L7Ae-CS3 experiment in the absence (dEGFP repressed) or presence (dEGFP unrepressed) of TEVp. BF: bright field. mKate: transfection marker used in the experiments. Scale bars indicate 200 μm.

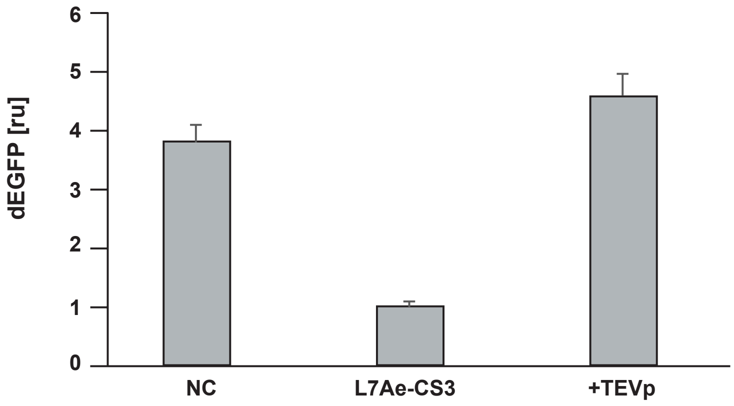

Supplementary Figure 4. Long-term repression of EGFP by L7AeCS3 and derepression by TEVp. To test the robustness of RNA translation regulation we evaluated EGFP levels 96h post-transfection. We observe sustained inhibition of EGFP translation in the presence of L7AeCS3, which is derepressed by TEVp expression. Data represent geometric mean and standard deviation of EGFP normalized by transfection marker mKate for n=3 technical replicates. NC: negative control. ru, relative units

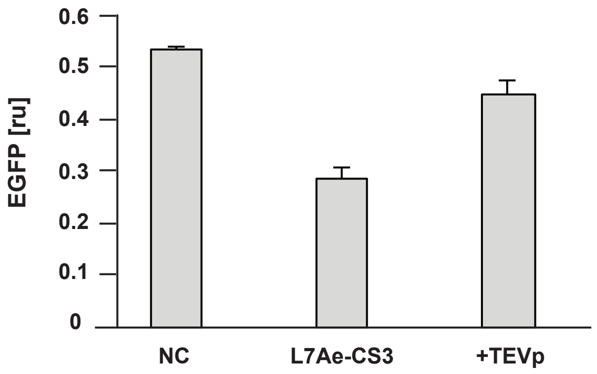

Supplementary Figure 5. Repression of EGFP by L7AeCS3 and derepression by TEVp in HeLa cells. EGFP levels were evaluated 48h post-transfection with flow cytometry, using mKate as transfection marker. We observe inhibition of EGFP translation in presence of L7AeCS3, and derepression in presence of TEVp co-expression. Data represent geometric mean and standard deviation of EGFP normalized by transfection marker mKate for n=2 technical replicates. NC: negative control. ru, relative units

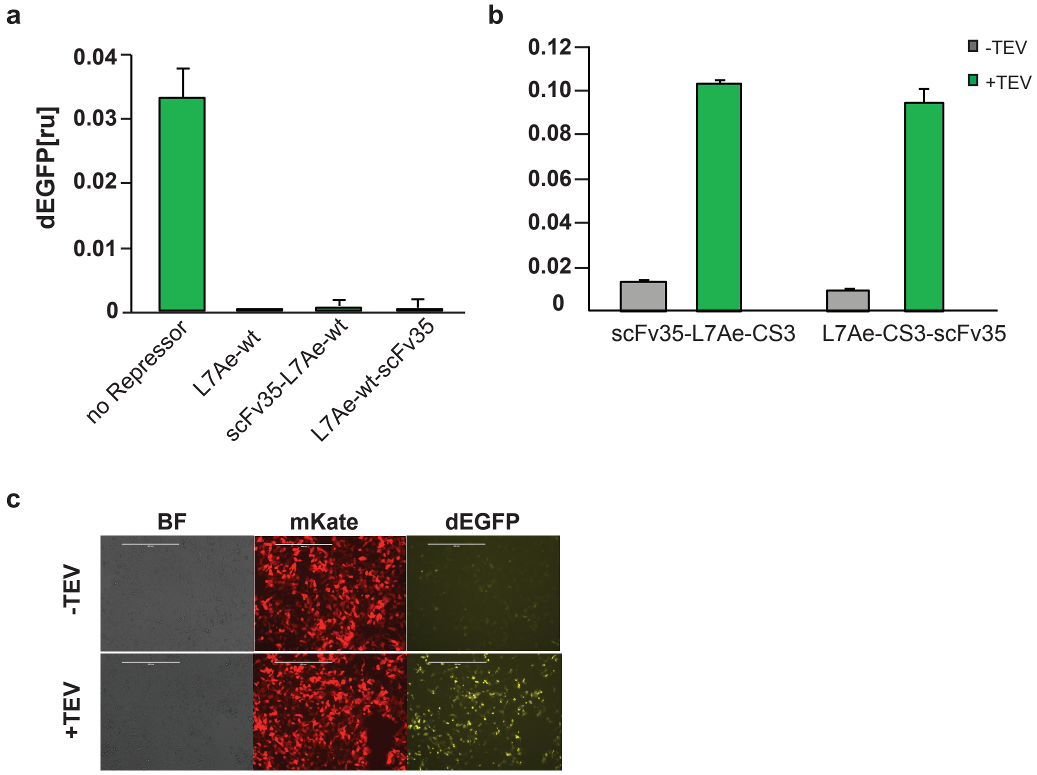

Supplementary Figure 6. Test of repression efficiency of wildtype L7Ae (L7Ae-wt) **(a)** and L7Ae-CS3 **(b)** fused to the N- or C-terminus of scFv35 intrabody. **(a)** Both scFv35-L7Ae-wt and L7Ae-wt-scFv35 show similar repression compared to L7Ae-wt alone. **(b)** In presence of TEVp, dEGFP levels increase, indicating inhibition of L7Ae-CS3 repression. Data collected 48h post transfection represent geometric mean and standard deviation of EGFP normalized by transfection marker mKate for n=3 replicates. ru, relative units. **(c)** Microscopy images for scFv35-L7Ae-CS3 experiment in presence or absence of TEVp. Scale bars indicate 200 μm. BF: bright field.

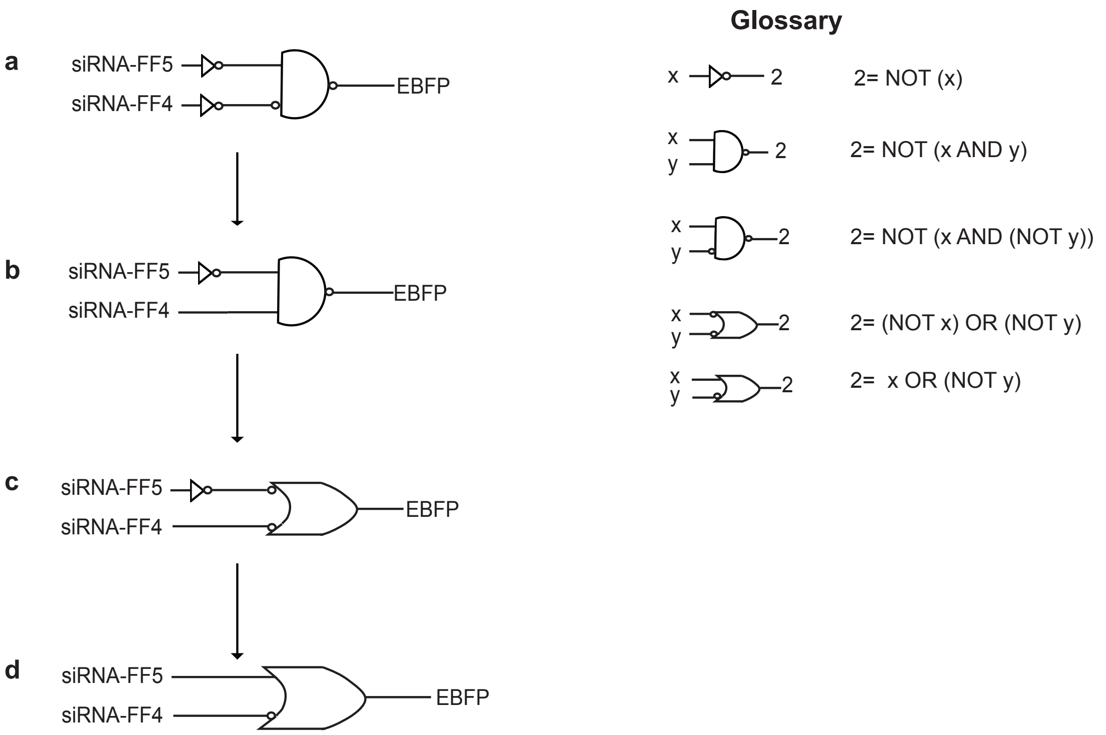

Supplementary Figure 7. Schematics of logic circuit simplification for Figure 2a. The logic function is computed from: EBFP = NOT (L7Ae); L7Ae = (NOT (siRNA-FF5)) AND (NOT TEVp); TEVp = (NOT siRNA-FF4). (**a**) Coalescing these terms yields: EBFP = NOT ((NOT (siRNA-FF5)) AND (NOT (NOT siRNA-FF4))). **(b)** Then, with elimination of NOT/NOT, it can be simplified to: EBFP = NOT (NOT (siRNA-FF5)) AND siRNA-FF4). **(c)** Further simplification using DeMorgan's law, where NOT (a AND b) = (NOT a) OR (NOT b) yields: EBFP = (NOT (NOT (siRNA-FF5))) OR (NOT siRNA-FF4). **(d)** And again, elimination of NOT/NOT finally results in: EBFP = siRNA-FF5 OR (NOT siRNA-FF4)

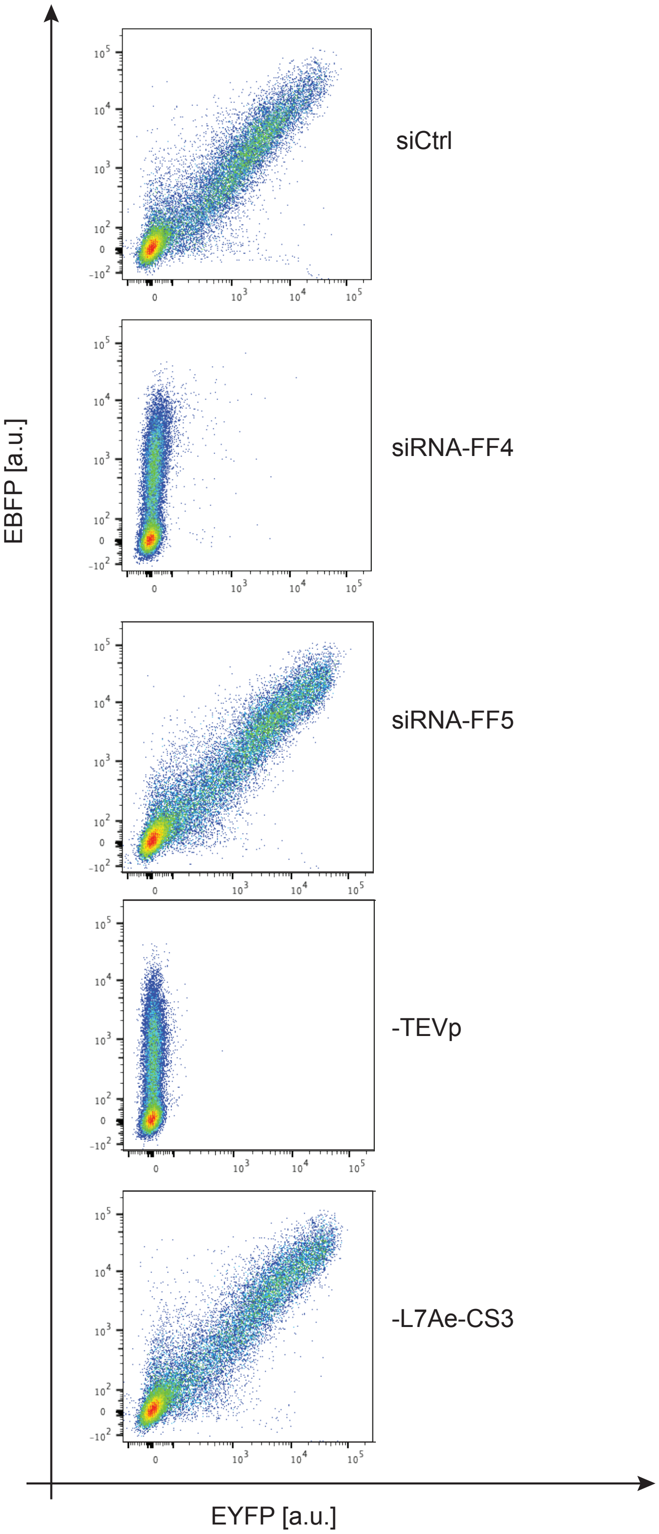

Supplementary Figure 8. Representative flow cytometry data for switch circuit (**Figure 2 c-d**). Populations of live, single cells were first determined based on forward and side scatter. Represented plots were established based on negative control (non-transfected) cells and cells transfected with EGFP, EBFP or mKate (transfection marker) only (not shown). EYFP is coupled to TEVp levels via a 2A self-cleaving peptide. EBFP is the functional output of L7Ae-CS3 activity. siRNAFF4 and siRNAFF5 are used to set the state of the switch (schematics is shown is **Figure 2c**).

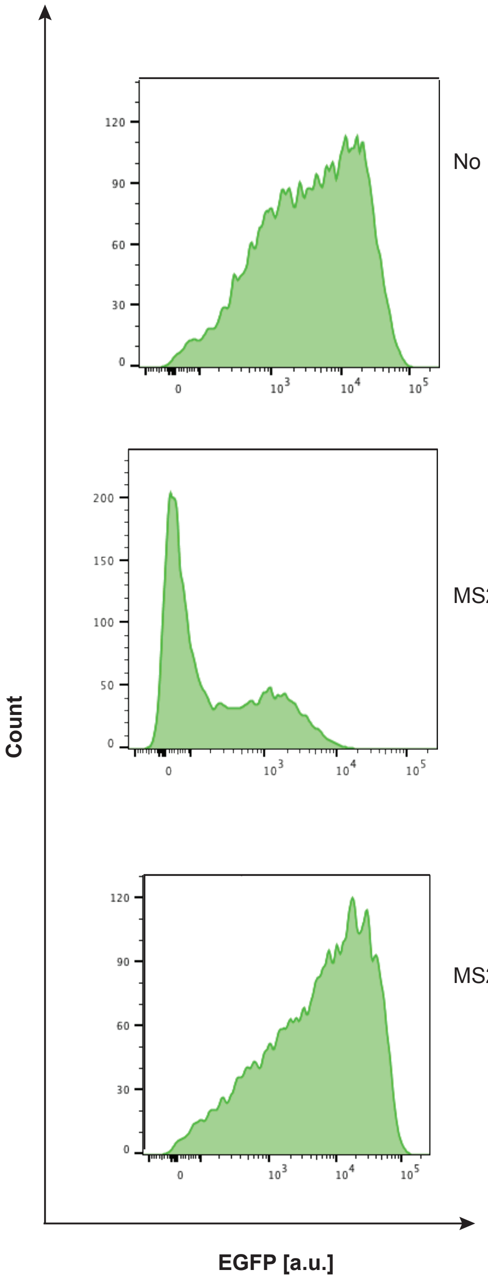

Supplementary Figure 9. Representative flow cytometry histograms of HEK293FT cells tested with MS2-TCS-cNOT7 regulated by TEVp **(Fig 3b).** Reporter EGFP includes 8x binding sites for MS2 in the 3'UTR, where MS2-TCS-cNOT7 binding induces mRNA degradation due to cNOT7 deadenylation activity. In the presence of TEVp, MS2-TCS-cNOT is cleaved and EGFP is derepressed.

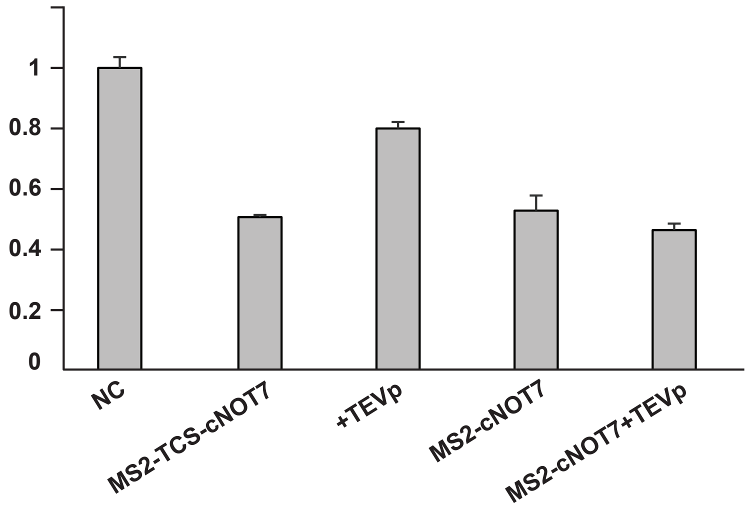

Supplementary Figure 10. Long-term repression of EGFP by MS2-CS-cNOT7 and derepression by TEVp. We evaluated EGFP levels 96h post-transfection in HEK293FT cells. We observe sustained inhibition of EGFP translation in presence of MS2-TCS-cNOT7, and increase in fluorescence levels upon TEVp co-expression. MS2-TCS-cNOT7 show similar repression to wild type MS2-cNOT7. MS2-cNOT7 activity is not affected by coexpression of TEVp. Data represent geometric mean and standard deviation EGFP normalized by transfection marker mKate for n=3 technical replicates. NC: negative control. ru, relative units

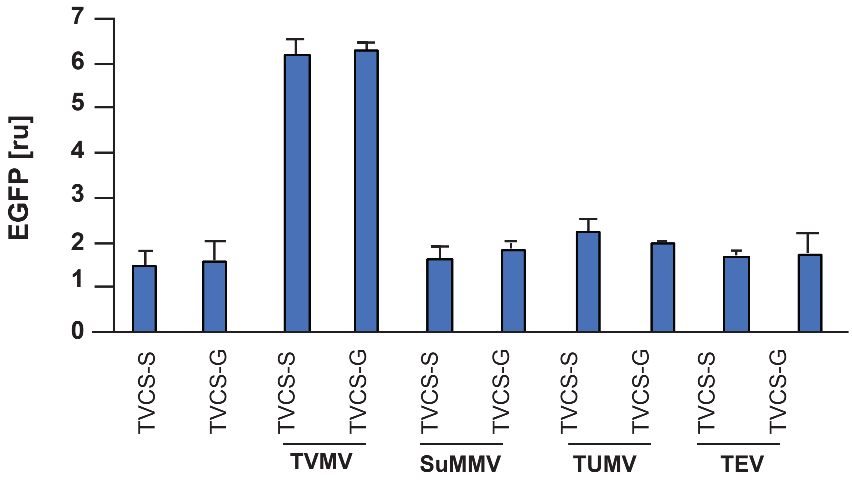

Supplementary Figure 11. Test of engineered MS2-TVCS/G-cNOT7 with cleavage site for TVMVp, either in the absence (first two columns on the left) or in presence of TVMVp, SuMMVp, TUMVp or TEVp. EGFP is repressed by MS2-TVCS/G-cNOT7, and TVMVp cleavage of the RBP results in increased EGFP fluorescence. Other proteases do not affect EGFP expression, indicating cleavage specificity. Data collected 48h post transfection represent geometric mean and standard deviation of EGFP normalized by transfection marker mKate for n=3 replicates. ru, relative units.

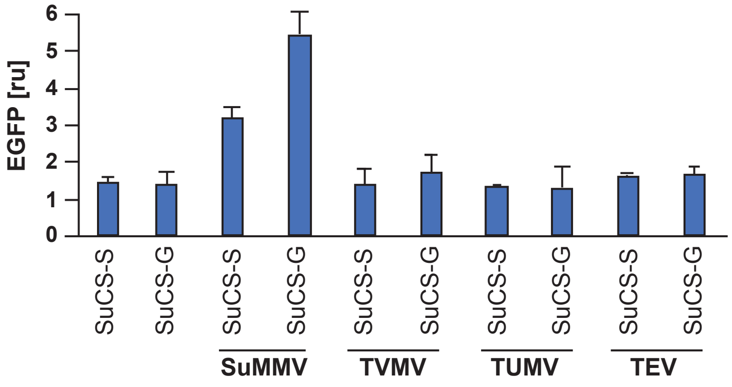

Supplementary Figure 12. Test of engineered MS2-SuCS/G-cNOT7 with cleavage site for SuMMVp, in the absence (first two columns on the left) or presence of SuMMVp, TVMVp, TUMVp or TEVp. Data indicate a specificity of SuMMVp toward cognate cleavage sequence, with higher EGFP upregulation with SuCS-G (Glycine in P1) rather than SuCS-S (Serine in P1). Data collected 48h post transfection represent geometric mean and standard deviation of EGFP normalized by transfection marker mKate for n=3 replicates. ru, relative units.

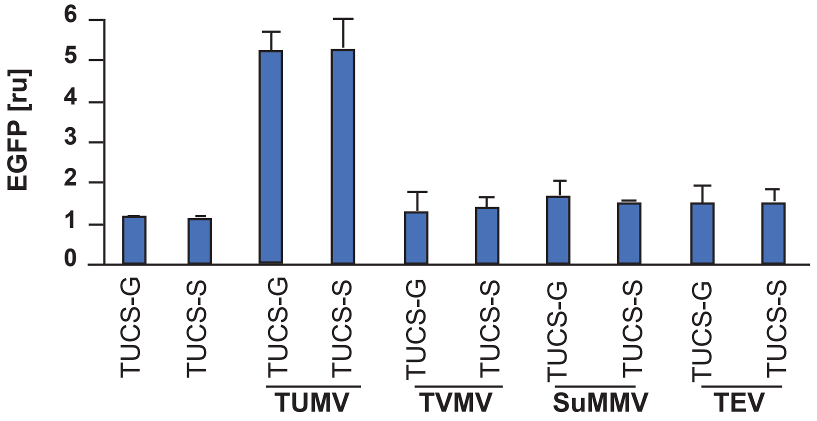

Supplementary Figure 13. Test of engineered MS2-TUCS/G-cNOT7 with cleavage site for TUMVp, in the absence (first two columns on the left) or presence of TUMVp, TVMVp, SuMMVp, TEVp. EGFP is de-repressed by TUMVp in a specific fashion. Data collected 48h post transfection represent geometric mean and standard deviation of EGFP normalized by transfection marker mKate for n=3 replicates. ru, relative units.

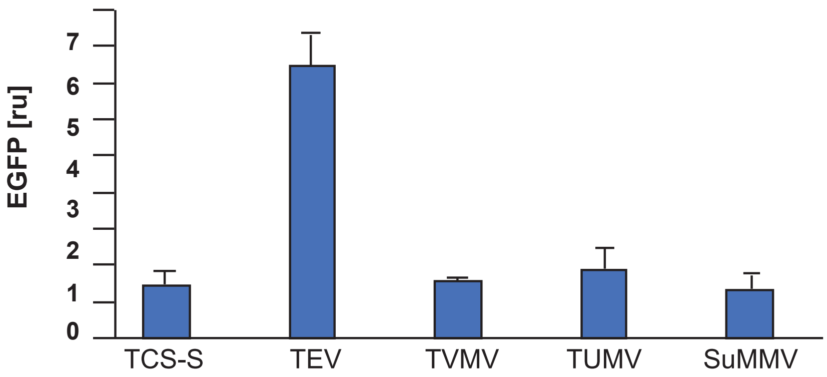

Supplementary Figure 14. Test of engineered MS2-TCS-cNOT7 with cleavage site for TEVp, in the absence of TEVp (first column on the left) or presence of TEVp, TVMVp, TUMVp, or SuMMVp. EGFP is efficiently de-repressed by TEVp in an orthogonal fashion. Data collected 48h post transfection represent geometric mean and standard deviation of EGFP normalized by transfection marker mKate for n=3 replicates. ru, relative units.

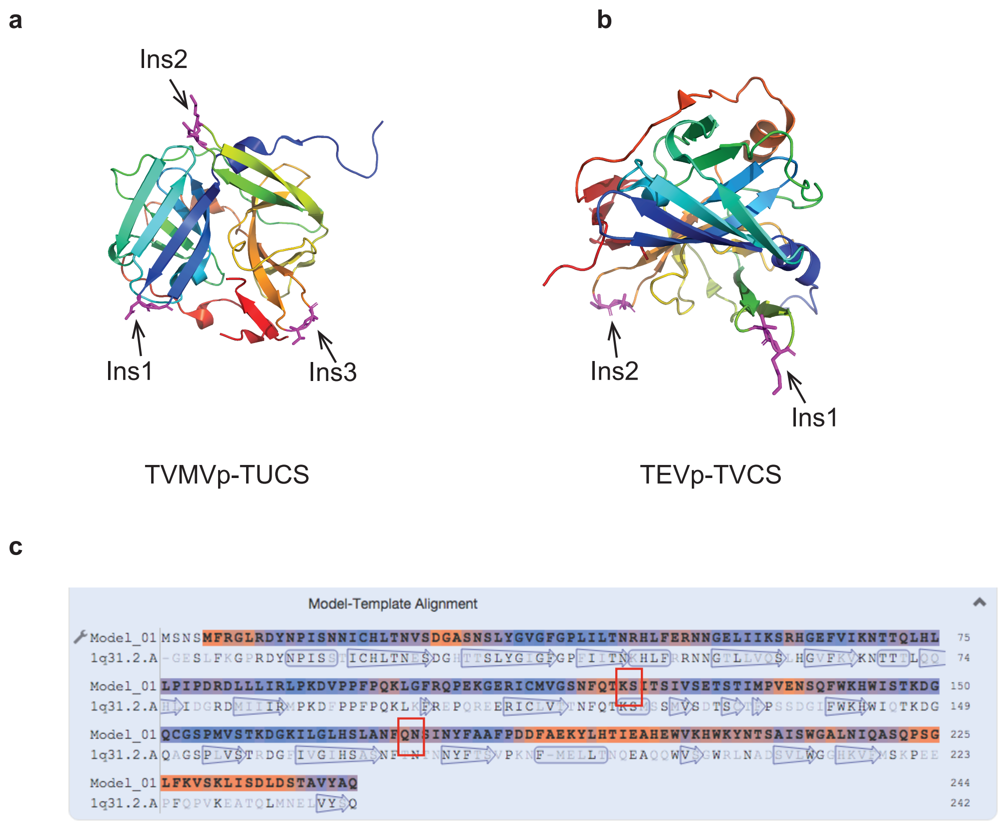

TEVp: 1q31
TUMVp: Model_01

Supplementary Figure 15. Engineered proteases with cleavage sites. **(a)** TVMVp with three alternative insertion points for TUMV cleavage site (TUCS). Insertion were placed between amino acid residues D26-G27 (Ins1, TVMVp-TUCS1), Q119-K120 (Ins2, TVMVp-TUCS2), T173-N174 (Ins3, TVMVp-TUCS3). **(b)** TEVp with insertion sites for TVMVp (TVCS). Insertions were placed between amino acid residues T117 and K118 (Ins1, TEVp-TVCS1) or T173-N174 (Ins2, TEVp-TVCS2). Protease structure visualization was performed with pymol (http://www.pymol.org). **(c)** TUMV structure was homology modeled using swiss model and TEV2 1Q31.pdb template. Shown is model-template alignment of TUMV sequence aligned to 1Q31 with insertion sites highlighted in red (Ins1:TK, Ins2:QN)

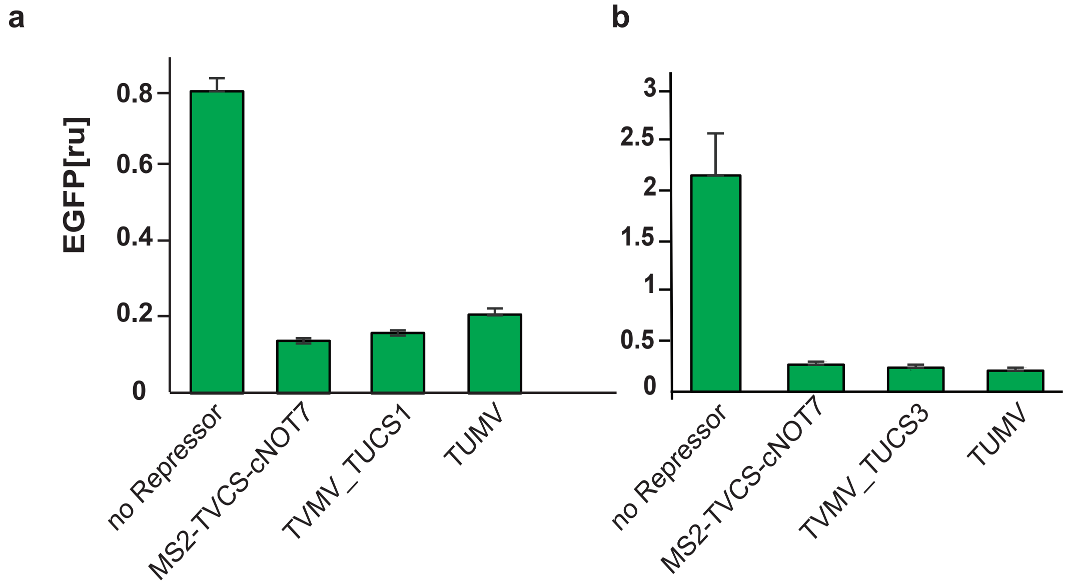

Supplementary Figure 16. Test of engineered TVMVp harboring cleavage sites for TUMVp (TUCS). Schematics of the cascade is shown in **Figure 4a.** In (**a**) TVMV includes the TUCS between amino acidic residues D26-G27, whereas in (**b**) the TUCS is placed between amino acidic residues T173-N174. Data show that both variants are not able to disrupt MS2-TVCS-cNOT7 function, perhaps because the insertions cause a loss-of-function modification of TVMVp structure. In **Figure 4b** we also show a variant, TVMV-TUCS2, that retains the ability to cleave MS2-TVCS-cNOT7 and is inhibited by TUMVp cleavage. Data collected 48h post transfection represent geometric mean and standard deviation of EGFP normalized by transfection marker mKate for n=3 replicates. ru, relative units.

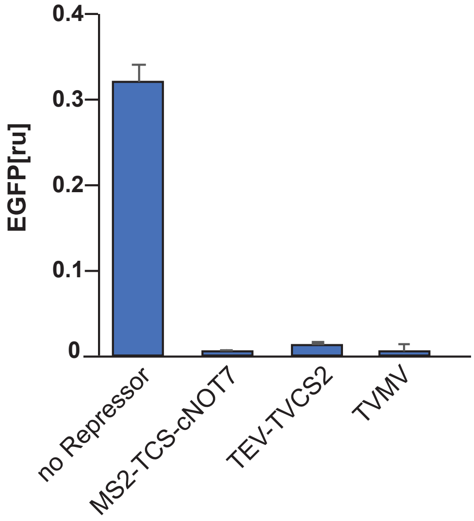

Supplementary Figure 17. Test of engineered TEVp harboring cleavage sites for TVMVp (TVCS) in HEK293FT cells. Schematics of the cascade is shown in **Figure 4c**. TVMVp cleavage site is inserted within T173-N174 residues of TEVp. Data show that TEV-TVCS2 is not able to rescue EGFP expression, indicating that similarly to TVMV-TUCS3, the insertion induces a structural modification of TEVp. Data collected 48h post transfection represent geometric mean and standard deviation of EGFP normalized by transfection marker mKate for n=3 replicates. ru, relative units.

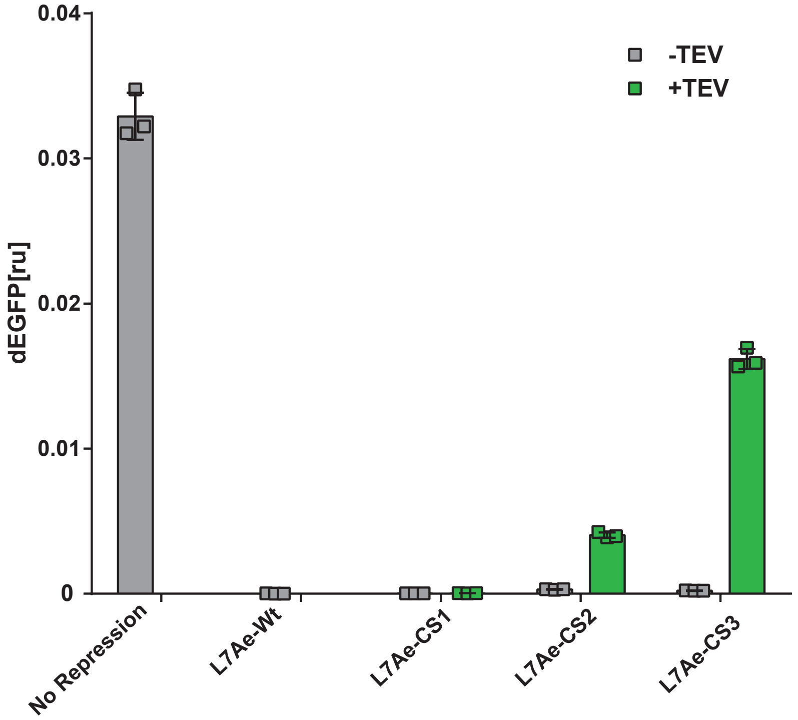

Supplementary Figure 18. Overlaid dot plot and bar chart for Fig 1c. Data represent geometric mean and standard deviation of dEGFP normalized by transfection marker mKate. n=3 replicates

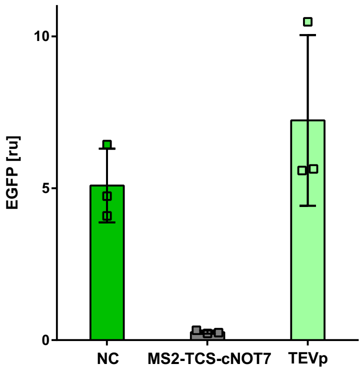

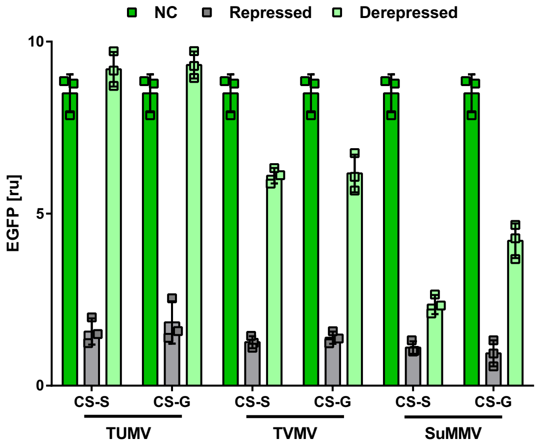

Supplementary Figure 19. Overlaid dot plot and bar chart for Fig 3b,d. Data represent geometric mean and standard deviation of EGFP normalized by transfection marker mKate for n=3 replicates.

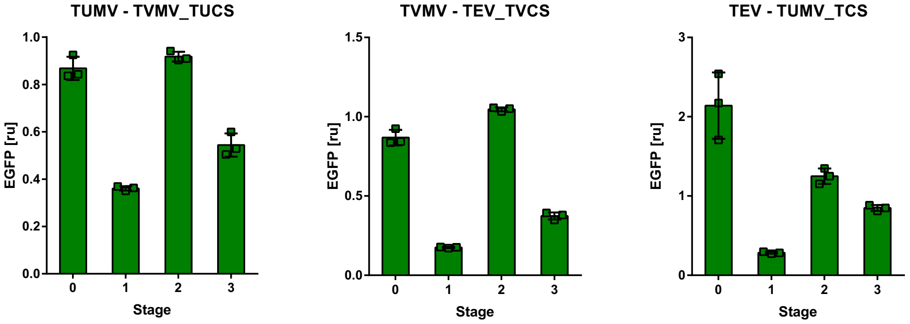

Supplementary Figure 20. Overlaid dot plot and bar chart for Fig 4b,d,e. Data represent geometric mean and standard deviation of EGFP normalized by transfection marker mKate for n=3 replicates.

Supplementary Note 1: Engineering a novel protease-based protein-protein regulation system.

We hypothesized that re-engineering proteases to include protease cleavage sites could provide for novel protein-protein system. Towards this goal, we first designed TVMVp with three alternative insertion sites for the TUMVp cleavage site and tested TVMV-CS along with MS2-TVCS-cNOT7 (MS2-cNot7 with cleavage site responsive to TVMVp) which in turn represses EGFP translation. In this topology, TVMV-CS upregulates EGFP expression, whereas co-expression of TUMVp results in TVMV-CS inhibition and EGFP down-modulation by MS2-TVCS-cNOT7 (**Figure 4a**).

Our data show that TVMVp_TUCS2 expression interferes with MS2-TVCS-cNOT7 activity, while expression of TUMVp in turn inhibits TVMVp_TUCS2, resulting in decreased EGFP expression (**Figure 4b**). Conversely, we did not observe inhibition of MS2-TVCS-cNOT7 by TVMVp_TUCS1 and TVMVp_TUCS3, perhaps because the insertions alter the structure and function of the proteins (**Supplementary Figure 11a,b**).

TEVp and TVMVp share a high degree of structural similarity, the crystal structure is resolved and for both[3,4]. Importantly, the amino acid sequence flanking the insertion point of TVMVp-TUCS2 (residues 116-121 NFQQ-KS) is highly similar to TEVp (amino acid residues 114-119 NFQT-KS). We thus inserted the TVMV-cleavage site (TVMV-CS) in the TEVp structure within amino acid residues T117 and K118 (TEVp-TVCS1) (**Figure 4c, Supplementary Figure 10b**). We also designed an additional variant with the cleavage site inserted within T173-N174 residues (TEVp-TVCS2). TEVp-TVCS1 efficiently impairs MS2-TCS-cNOT7 repression of EGFP, whereas addition of TVMVp inhibits TEVp-TVCS1 function (**Figure 4d**). Conversely, similar to TVMV-TUCS3 protease, we did not observe activity for TEVp-TVCS2 (**Supplementary Figure 12**). Our results indicate that the best insertion site within these proteases is the NFQX-KS, where X refers to variable amino acid residue. Based on this information, we designed a structurally modified TUMVp with TEVp cleavage site insertion (TCS) (**Figure 4e**). Importantly, the TUMVp crystal structure is not resolved, but it is homologous to TEVp (**Supplementary Figure 10c**). We then included a TCS between amino acid residues T116 and K117 of TUMVp (NFQX-KS consensus), while MS2-TUCS-cNOT7 regulates EGFP translation (**Figure 4e**). Our data indicate that TUMV-TCS inhibits MS2-TUCS-cNOT7, and TEVp reverts this effect, albeit with reduced efficiency compared to the other proteases (**Figure 4f**).

Supplementary Table 1. List of the plasmids used in this study. Plasmids sequences are available on GenBank.

Fig.	Short plasmid name	Full plasmid name	Parts from	GenBank accession code
1,3&SF2,3,6,7,8	p96	pGTW6-hEF1a-TEVp	Siciliano et al[7]	MH883330
1	p97	pGTW6-CMV-L7Ae-CS1	Wroblewska et al[5], Siciliano et al[7]	MH883331
1	p98	pGTW6-CMV-L7Ae-CS2	Wroblewska et al[5], Siciliano et al[7]	MH883332
1,2&SF2,3	p99	pGTW6-CMV-L7Ae-CS3	Wroblewska et al[5], Siciliano et al[7]	MH883333
1&SF2,4	pL-R6	pT-GTW6-CMV-L7Ae	Wroblewska et al[5], Addgene id 59560	
1	p37	pEXPR-2_3-TRE_tight-TEVp-LD0-scFv162	Siciliano et al[7]	MH107780
1	pCMV-rtTA3	pCMV-rtTA3	Siciliano et al[7]	Clonetech
SF4	p124	pGTW6-CMV-scFv35-L7Ae	Wroblewska et al[5]	MH883335
SF4	p125	pGTW6-CMV-L7Ae-scFv35	Wroblewska et al[5]	MH883336
1& SF5	p143	pGTW6-CMV-scFv35-L7AeCS3	Wroblewska et al[5], Siciliano et al[7]	MH883339
1&SF5	p146	pGTW6-CMV-L7AeCS3-scFv35	Wroblewska et al[5], Siciliano et al[7]	MH883340
1	p16	pEXPR3_4-Hef1a-nNS3	Siciliano et al[7]	MH107778
Ctrl	pL-A1	pT-GTW6-CMV-mKate	Siciliano et al[7]	MH107777
1&SF2,3	pL-S18	pBoxCDGC_2xKMet_DD-EGFP	pL-S1, Wroblewska et al[5], Addgene id 59564	

2	p131	pGTW6-CMV-TEVp-P2A-EYFP-4XFF4	Wroblewska et al[5], Siciliano et al[7]	MH883357
2&SF5	p122	pBoxCDGC_2xKMet-EBFP2	pL-S1, Wroblewska et al[5]	MH883334
2&SF5	p127	pBoxCDGC_2xKMet-TEVp-P2A-EYFP-4XFF4	pL-S1, Wroblewska et al[5], Siciliano et al[7]	MH883337
3	pL-R1	pT-GTW6-CMV-MS2-CNOT7	Wroblewska et al[5]	MH883359
3	P133	pT-GTW6-CMV-L7AeCS3-4xFF5	Wroblewska et al[5]	MH883360
3&SF9	p134	pGTW6-CMV-MS2-TCS(S)-CNOT7-P2A-EBFP2-4XFF5	Wroblewska et al[5], Siciliano et al[7]	MH883338
3,4&SF6-9,11	pL-C1	pBoxCDGCmut_KMet-EGFP-8xMS2-pA	Wroblewska et al[5]	MH883358
3&SF7	p147	pGTW6-CMV-MS2-SUMMV_CS(S)-CNOT7-P2A-EBFP2-4XFF5	Wroblewska et al[5], Jesus Fernandez-Rodriguez[6]	MH883341
3&SF7	p148	GTW6-CMV-MS2-SUMMV_CS(G)-CNOT7-P2A-EBFP2-4XFF5	Wroblewska et al[5], Jesus Fernandez-Rodriguez[6]	MH883342
3&SF8	p149	pGTW6-CMV-MS2-TUMV_CS(G)-CNOT7-P2A-EBFP2-4XFF5	Wroblewska et al[5], Jesus Fernandez-Rodriguez[6]	MH883343
3&SF8	p150	pGTW6-CMV-MS2-TUMV_CS(S)-CNOT7-P2A-EBFP2-4XFF5	Wroblewska et al[16], Jesus Fernandez-Rodriguez[18]	MH883344
3&SF6	p151	pGTW6-CMV-MS2-TVMV_CS(G)-CNOT7-P2A-EBFP2-4XFF5	Wroblewska et al[5], Jesus Fernandez-Rodriguez[6]	MH883345
3&SF6,11	p152	pGTW6-CMV-MS2-TVMV_CS(S)-CNOT7-P2A-EBFP2-4XFF5	Wroblewska et al[5], Jesus Fernandez-Rodriguez[6]	MH883346

3&SF6-9,12	p153	pGTW6-CMV-TVMV	Jesus Fernandez-Rodriguez[6]	MH883347
3&SF6-9	p154	pGTW6-CMV-SUMV	Jesus Fernandez-Rodriguez[6]	MH883348
3&SF6-9,11	p155	pGTW6-CMV-TUMV	Jesus Fernandez-Rodriguez[6]	MH883349
4	pE1	pGTW6_CMV-TEV2-TVMV_CS1	Wroblewska et al[5], Siciliano et al[7]	MH883350
4,12	pE2	pGTW6_CMV-TEV2-TVMV_CS2	Wroblewska et al[5], Siciliano et al[7]	MH883351
4	pE3	pGTW6_CMV-TVMV-TUMV_CS2	Jesus Fernandez-Rodriguez[6]	MH883352
4	pE4	pGTW6_CMV-TEV2	Wroblewska et al[5], Siciliano et al[7]	MH883353
4	pE5	pGTW6-CMV-TUMV-TEV-CS1	Wroblewska et al[5], Siciliano et al[7]	MH883354
4,11	pE6	pGTW6_CMV-TVMV-TUMV_CS1	Jesus Fernandez-Rodriguez[6]	MH883355
4,11	pE7	pGTW6_CMV-TVMV-TUMV_CS3	Jesus Fernandez-Rodriguez[6]	MH883356

Supplementary Table 2. List of the primers used to generate MS2-cNOT7 and orthogonal proteases with cleavage insertion sites.

Construct	Primer name	Primer Sequence
MS2-CS-cNOT7	1-Fw MS2 overhang infusion (common to all cloning)	CATTTCAGGTGGATCCGCCACCATGGCTTCTAACTTTACTCAG
MS2-CS-cNOT7	2-Rv cNOT7 overhang infusion (common to all cloning)	GCCAACAAGCAGTCAGTTTAATTAAAGCTAGTTACCC
MS2-TCS-S)-cNOT7	3-Rv MS2	GGACTGGAAGTACAGGTTTTCGGCGATCGAGTAGATGCCGGAGTTTGCTGC
MS2-TCS-S)-cNOT7	4-Fw cNOT7	CTGTACTTCCAGTCCGGCATGCCAGCGGCAACTGTAGAT
MS2-SuCS-S-cNOT7	5-Rv MS2	GCCGGACTGCAGATGGATTTCCTCGGCGATCGAGTAGATGCCGGAGTTTGCT
MS2-SuCS-S-cNOT7	6-Fw cNOT7	GAGGAAATCCATCTGCAGTCCGGCATGCCAGCGGCAACTGTAGA
MS2-SuCS-G-cNOT7	7-Rv MS2	GCCCTGCAGATGGATTTCCTCGGCGATCGAGTAGATGCCGGAGTTTGCTG
MS2-SuCS-G-cNOT7	8-Fw cNOT7	GAGGAAATCCATCTGCAGGGCATGCCAGCGGCAACTGTAGA
MS2-TUCS-S-cNOT7	9-Rv MS2	GCCGGACTGGTGATACACACAGGCGGCGATCGAGTAGATGCCGGAGTTTGCT
MS2-TUCS-S-cNOT7	10-Fw cNOT7	GCCTGTGTGTATCACCAGTCCGGCATGCCAGCGGCAACTGTAGA
MS2-TUCS-G-cNOT7	11-Rv MS2	GCCCTGGTGATACACACAGGCGGCGATCGAGTAGATGCCGGAGTTTGCT
MS2-TUCS-G-cNOT7	12-Fw cNOT7	GCCTGTGTGTATCACCAGGGCATGCCAGCGGCAACTGTAGA
MS2-TVCS-G-cNOT7	13-Rv MS2	GCCCTGAAACCGCACTGTCTCGGCGATCGAGTAGATGCCGGAGTTTGCT
MS2-TVCS-G-cNOT7	14-Fw cNOT7	GAGACAGTGCGGTTTCAGGGCATGCCAGCGGCAACTGTAGA

MS2-TVCS-S-cNOT7	15-Rv MS2	GCCGGACTGAAACCGCACTGTCTCGGCGATCGAGTAGATGCCGGAGTTTGCTGC
MS2-TVCS-S-cNOT7	16-Fw cNOT7	GAGACAGTGCGGTTTCAGTCCGGCATGCCAGCGGCAACTGTAGA
TEV_TVCS1	P1	CATTTCAGGTGGATCCCGCCACCATGAGCCTGTTCAAGGGCCCC
TEV_TVCS1	P2	GCCCTGAAACCGCACTGTCTCGGTCTGGAAGTTTGTTGTGAC
TEV_TVCS1	P3	GAGACAGTGCGGTTTCAGGGCAAGAGCATGAGCAGCATGGTG
TEV_TVCS1	P4	GGGTAACTAGCTTTAATTAATCACTGGCTGTACACCAGCTC
TEV_TVCS2	P1	CATTTCAGGTGGATCCCGCCACCATGAGCCTGTTCAAGGGCCCC
TEV_TVCS2	P5	GCCCTGAAACCGCACTGTCTCGGTGAAGTTGCTGGCGCTGTG
TEV_TVCS2	P6	GAGACAGTGCGGTTTCAGGGCAACACCAACAACTACTTCACC
TEV_TVCS2	P4	GGGTAACTAGCTTTAATTAATCACTGGCTGTACACCAGCTC
TVMV_TUCS1	P13	CATTTCAGGTGGATCCGCCACCATGTCTAAGGCCCTGCTGAAG
TVMV_TUCS1	P14	GCCCTGGTGATACACACAGGCATCGGAGCTGTTTTCCAGCAG
TVMV_TUCS1	P15	GTGTATCACCAGGGCGGCCACAGCGAGCGGCTGTTC
TVMV_TUCS1	P16	GGGTAACTAGCTTTAATTAATCATTCCACCAGGGTGAAGCTG
TVMV_TUCS2	P13	CATTTCAGGTGGATCCGCCACCATGTCTAAGGCCCTGCTGAAG
TVMV_TUCS2	P17	GCCCTGGTGATACACACAGGCCTGCTGGAAGTTGGTGGACAC
TVMV_TUCS2	P18	GTGTATCACCAGGGCAAAAGCGTGTCCAGCCTGGTG

TVMV_TUCS2	P16	GGGTAACTAGCTTTAATTAATCATTCCACC AGGGTGAAGCTG
TVMV_TUCS3	P13	CATTTCAGGTGGATCCGCCACCATGTCTAA GGCCCTGCTGAAG
TVMV_TUCS3	P19	GCCCTGGTGATACACACAGGCGGTGGTGT GGGTCAGGCTGTG
TVMV_TUCS3	P20	GTGTATCACCAGGGCAACGGCAGCAACTA CTTCGTG
TVMV_TUCS3	P16	GGGTAACTAGCTTTAATTAATCATTCCACC AGGGTGAAGCTG
TUMV-TCS	P7	CATTTCAGGTGGATCCGCCACCATGAGCA ACAGCATGTTCCGG
TUMV-TCS	P8	GGACTGGAAGTACAGGTTTTCGGTCTGGA AGTTGCTTCCCAC
TUMV-TCS	P9	CCTGTACTTCCAGTCCAAGAGCATCACCAG CATCGTG
TUMV-TCS	P10	GGGTAACTAGCTTTAATTAATTACTGGGCG TACACGGCGGTG

Supplementary Table 3. Transfection tables for all experiments in this study

Figure 1c

	pL-S18	pL-A1	p97/p98/p99	empty	p96
Repression	50 ng	60 ng	150 ng	140 ng	
Derepression	50 ng	60 ng	150 ng	80 ng	60 ng
Control	50 ng	60 ng		290 ng	
Control L7Ae-wt pL-R6	50 ng	60 ng	150ng	140 ng	
Reagent/cells					
DMEM	98ul				
Attractene	1.5 ul				
HEK 293FT	200,000				

Figure 1e

	p143/p146	pLS18	pL-A1	empty	p38	P16	CMV-rtTA
Repression	150 ng	50 ng	60 ng	60 ng	60 ng		60 ng
Derepression	150 ng	50 ng	60 ng		60 ng	60 ng	60 ng
Control	50 ng	60 ng		290 ng			
Reagent/cells							
DMEM	98ul						
Attractene	1.5 ul						
HEK 293FT	200,000						

Supplementary Figure 4a,b

	p124/p125	pLS18	pL-A1	empty	
Repression	150 ng	50 ng	60 ng	140 ng	
	p143/p146	pLS18	pL-A1	empty	p96
Repression	150 ng	50 ng	60 ng	140 ng	
Derepression	150 ng	50 ng	60 ng	80 ng	60
Control	50 ng	60 ng		290 ng	
Reagent/cells					
DMEM	98ul				
Attractene	1.5 ul				
HEK 293FT	200,000				

Figure 2b

	p122	p131	pL-A1	empty	p133
Stage 0	50 ng		50 ng	300 ng	
Stage 1	50 ng		50 ng	200 ng	100 ng
Stage 2	50 ng	100 ng	50 ng	100 ng	100 ng
Input 1 5pmol, FF5	50 ng		50 ng	200 ng	100 ng
Input 2 5pmol, FF4	50 ng	100 ng	50 ng	100 ng	100 ng
Reagent/cells					
DMEM	96ul				
Attractene	1.5 ul				
HEK 293FT	200,000				

Figure 2d

	p122	p126	pL-A1	empty	p133
siCtrl	50 ng	100 ng	50 ng	100 ng	100 ng
siRNA-FF4 5pmol, FF4	50 ng	100 ng	50 ng	100 ng	100 ng
siRNA-FF5 5pmol, FF5	50 ng	100 ng	50 ng	100 ng	100 ng
Control -TEVp	50 ng		50 ng	200 ng	100 ng

Control -L7Ae-CS3	50 ng	100 ng	50 ng	200 ng	
Reagent/cells					
DMEM	96ul				
Attractene	1.5 ul				
HEK 293FT	200,000				

Figure 3b Supplementary Figures 6-9,14

**Fig 3b**	p134	p96	pL-A1	empty	pL-C1	pR-1
NC			60 ng	280 ng	60 ng	
MS2-TCS-CNOT7	60 ng		60 ng	220 ng	60 ng	
TEVp	60 ng	60 ng	60 ng	160 ng	60 ng	
MS2-cNOT7			60 ng	220 ng	60 ng	60 ng
**Fig 3d**	p147/p148/p149/p150/151/p152	p154/p153/p155	pL-A1	empty	pL-C1	
-	60 ng		60 ng	220 ng	60 ng	
+	60 ng	60 ng	60 ng	160 ng	60 ng	
Reagent/cells						
DMEM	96ul					
Attractene	1.5 ul					
HEK 293FT	200,000					

Figure 4 b,c,d Supplementary Figure 11,12

	MS2-CS -CNOT7	Engineered protease	pL-A1	empty	pL-C1	Wild-type protease
Stage 0: No repression			30 ng	345 ng	25ng	
Stage1: Ms2-CS-cNOT7	50 ng		30 ng	295 ng	25 ng	
Stage2: Engineered protease	50 ng	30 ng	30 ng	265 ng	25 ng	
Stage3: Wild-type protease	50 ng	30 ng	60 ng	215 ng	25 ng	50 ng
Reagent/cells						
Optimem	96ul					
Lipofectiamine	1.5 ul					
HEK 293FT	130,000					

References

1. Moore, T., Zhang, Y., Fenley, M. O. & Li, H. Molecular basis of box C/D RNA-protein interactions; cocrystal structure of archaeal L7Ae and a box C/D RNA. *Structure* 12**,** 807–18 (2004).
2. The PyMOL Molecular Graphics System, Version 2.0 Schrödinger, LLC.
3. Phan, J. *et al.* Structural Basis for the Substrate Specificity of Tobacco Etch Virus Protease. J*ournal of Biological Chemistry* 277, 50564-50572 (2002).
4. Sun, P., Austin, B. P., Zsef, J., Zsé, T. & Waugh, D. S. Structural determinants of tobacco vein mottling virus protease substrate specificity. Protein Science 19, 2240-2251 (2010).
5. Wroblewska, L. et al. Mammalian synthetic circuits with RNA binding proteins for RNA-only delivery. *Nature Biotechnology* 33, 839-841 (2015).
6. Fernandez-Rodriguez, J. & Voigt, C. Post-translational control of genetic circuits using Potyvirus proteases. *Nucleic Acids Research* 44, 6493-6502 (2016).
7. Siciliano, V. et al. Engineering modular intracellular protein sensor-actuator devices. *Nature Communications* 9, (2018).
